# Drug Targeting of Inflammatory Bowel Diseases by Biomolecules

**DOI:** 10.3390/nano11082035

**Published:** 2021-08-10

**Authors:** Joana Costa Antunes, Catarina Leal Seabra, Joana Margarida Domingues, Marta Oliveira Teixeira, Cláudia Nunes, Sofia Antunes Costa-Lima, Natália Cândido Homem, Salette Reis, Maria Teresa Pessoa Amorim, Helena Prado Felgueiras

**Affiliations:** 1 Centre for Textile Science and Technology (2C2T), Campus de Azurém, University of Minho, 4800-058 Guimarães, Portugal; joana.domingues@2c2t.uminho.pt (J.M.D.); pg35037@alunos.uminho.pt (M.O.T.); natalia.homem@2c2t.uminho.pt (N.C.H.); mtamorim@det.uminho.pt (M.T.P.A.); helena.felgueiras@2c2t.uminho.pt (H.P.F.); 2 Laboratório Associado para a Química Verde (LAQV), Network of Chemistry and Technology (REQUIMTE), Departamento de Ciências Químicas, Faculdade de Farmácia, Universidade do Porto, 4050-313 Porto, Portugal; cseabra@ff.up.pt (C.L.S.); cdnunes@ff.up.pt (C.N.); slima@ff.up.pt (S.A.C.-L.); shreis@ff.up.pt (S.R.)

**Keywords:** biologics, gut dysbiosis, infection, microbiota, Crohn’s disease, ulcerative colitis, treatment, polymeric nanoparticles

## Abstract

Inflammatory bowel disease (IBD) is a group of disabling, destructive and incurable immune-mediated inflammatory diseases comprising Crohn’s disease (CD) and ulcerative colitis (UC), disorders that are highly prevalent worldwide and demand a large investment in healthcare. A persistent inflammatory state enables the dysfunction and destruction of healthy tissue, hindering the initiation and endurance of wound healing. Current treatments are ineffective at counteracting disease progression. Further, increased risk of serious side effects, other comorbidities and/or opportunistic infections highlight the need for effective treatment options. Gut microbiota, the key to preserving a healthy state, may, alternatively, increase a patient’s susceptibility to IBD onset and development given a relevant bacterial dysbiosis. Hence, the main goal of this review is to showcase the main conventional and emerging therapies for IBD, including microbiota-inspired untargeted and targeted approaches (such as phage therapy) to infection control. Special recognition is given to existing targeted strategies with biologics (via monoclonal antibodies, small molecules and nucleic acids) and stimuli-responsive (pH-, enzyme- and reactive oxygen species-triggered release), polymer-based nanomedicine that is specifically directed towards the regulation of inflammation overload (with some nanosystems additionally functionalized with carbohydrates or peptides directed towards M1-macrophages). The overall goal is to restore gut balance and decrease IBD’s societal impact.

## 1. Introduction to Inflammatory Bowel Diseases (IBD)

Inflammatory bowel diseases (IBD) are disabling, noncommunicable, progressive and incurable immune-mediated inflammatory diseases (IMIDs). Crohn’s disease (CD) and ulcerative colitis (UC) constitute the most prevalent forms of IBD. These diseases are highly prevalent worldwide, particularly in Europe and North America, and are spreading globally at an accelerated rate [1,2]. IBD is most frequent in young females, especially those of approximately 30 years of age. However, the incidence of IBD is also increasing among young males, with early-onset IBD (age below 18 years old) forming as a major contributor to IBD conservancy [2,3]. In Portugal, data from 2020 estimate that approximately 150 IBD patients exist per 100,000 inhabitants, with equal distribution between CD and UC, and with CD mainly occurring in females ranging from 17 to 39 years old and UC occurring in patients from 40 to 64 years old [2,4].

These lifelong diseases typically encircle remission and relapse cycles, with shorter remission periods and longer and intensified relapse stages as the disease progresses. This, associated with the direct cost of medication, hospitalization, and therapeutic interventions such as surgeries; indirect charges subsequent to the loss of productivity, unemployment, absenteeism, early retirement, or death; and costs to both IBD patients and their caregivers, including travel expenses, time off from work, and non-refundable treatment costs, are of particular concern given the exponentially growing impaired IBD population and disease severity [2,5,6].

A westernized lifestyle, urbanization and industrialization are known as the driving forces of IBD initiation and endurance [2]. Regardless, IBD arises from intricate exchanges between host genetics, intestinal barrier function, the immune system, environmental factors, and the gut microbiome [5,6]. The cause remains unknown, but it appears to occur in individuals carrying specific genetic alterations, which develop an atypical immune response to certain bowel pathobionts following interaction with exacerbating environmental factors [6,7,8].

A genetic predisposition to IBD pathogenesis, namely, the existence of a family member with IBD, is strongly connected to a higher risk of CD or UC materialization. Siblings of CD patients have from a 2–15% to 25–35% greater chance of developing this disorder than people with no familial links to the disease [9]. To date, 242 gene variants have been associated with IBD. Statistically significant causal variants include *ATG16L1*, *CDH1*, *HNF4α*, *IL10*, *IL10RA*, *IL10RB*, *IL23R*, *IRGM*, *LRRK2*, *NOD2* and *PTPN2*, genes that are linked to innate mucosal defence, Paneth cells, autophagy, epithelial barrier, immune cell recruitment, antigen presentation, regulatory T (Treg) cells, immune tolerance, endoplasmic reticulum stress, oxidative stress and cell apoptosis [6,10].

The intestinal mucosal barrier (with innate immune cells, epithelial cells (IECs), intraepithelial lymphocytes and the mucosal lining) constitutes the front wall, which is encountered by food antigens and intestinal commensal and pathogenic microorganisms, working with the intestinal luminal contents to maintain homeostasis [5]. Upon facing IBD, this barrier is compromised. Increased barrier permeability for dietary components and gut microbiota, inhibition of epithelium matrix remodeling and regeneration and antimicrobial activity, and a decrease in intracellular pathogen clearance and amplification of intestinal inflammation are known consequences [7,9,10,11].

IBD patients sustain multiple deviations from a normal inflammatory response to an antigen or cytokine [7,8,12,13]. Neutrophils, dendritic cells, macrophages, and innate lymphoid cells reinforce the intestinal mucosal barrier at the first level of defence of the mucosal innate immune system [14,15]. In healthy individuals, intestinal macrophages show attenuated proliferative rates and chemotactic abilities, while retaining the phagocytic and bactericidal function, effectively regulating adaptative T cell responses. Pathogenic Th1 and Th17 responses are restrained, and tolerogenic Treg cells are stimulated [15]. In IBD, a defective secretion of pro-inflammatory cytokines impairs neutrophil recruitment and pathogen clearance. Chronic inflammation occurs, and excessive pro-inflammatory cytokine production (e.g., TNF-α, IL-12, IL-17 and IL-23) includes an exaggerated and intolerant T cell-induced response, unrestrained inflammation, and aggravated intestinal bowel damage [12,14,15,16,17]. Treg presence is reduced [12].

The socio-environmental factors influence IBD-associated cases at an (i) individual level: living habits (smoking—CD-exclusive, hygiene status), diet (poor in plant-based fibres), null or scarce physical exercise, psychological stress, medical history (childhood infections and vaccination, early-life antibiotic use, frequent intake of oral contraception and nonsteroidal anti-inflammatory agents, vitamin D deficiency, appendectomy), breastfeeding, etc.; (ii) population-level health interventions, such as differentiated access to healthcare and fluctuating IBD presence in migrants, among others. Physical components, with the air or water as vehicles of contaminants, such as: (i) air pollution or specific contaminants that can relate to the living areas or vehicle use; (ii) water contamination derived from deficient access to tap or hot water, leaching from pipes, food adjuvants, or by-products of industrial activities [3,10,18,19].

The human microbiome is constituted of a complex network of co-existing microbes, particularly within the human intestine [20]. Its composition varies throughout life, and intestinal homeostasis is preserved in healthy individuals [21]. However, failure to reach host–microbiota equilibrium can create a dysbiosis, inducing changes in gut microbial diversity and an inbalance between commensal and pathogenic microorganisms [22], which is linked to IBD susceptibility, onset and/or aggravation [21,23]. Prolonged gut dysbiosis enables pathogens to dominate the gut and favours the propagation of pro-IBD species, in parallel with the eradication of anti-IBD microbes [10,21,23].

## 2. Gut Microbiota and IBD Progression

The resident gut microbiota are involved in several vital host physiological processes, including the development of the gut immune system, digestion of dietary factors, and colonization resistance against incoming pathogens, but it can also be associated with UC and CD pathogenesis [24,25]. Microbial antigens and their metabolic products are key promoters of barrier dysfunction in IBD, with a higher concentration of anaerobic bacteria in the distal ileum and colon, encouraging the appearance of IBD [22,25]. However, the presence of specific pathobionts within the bowel, and their correlation with the onset of IBD, remains unclear [22]. An imbalance in gut microbiota results in a change in the gut microflora-associated functions, such as changes in fermentation products, mainly carbohydrate, vitamins, and short-chain fatty acids (SCFA), and changes in biochemical processes, such as immune equilibrium imbalance [26]. Dysbiosis has been described as the root of IBD etiopathology, with differences between healthy and diseased gut microbiota regarding diversity and number [26,27,28]. For instance, Britton et al. showed that these microorganisms can modulate the immune system, namely, microbiota-specific anti- and pro-inflammatory activity. Anti-inflammatory RORγt^+^ Treg cells are microbiota-dependent and are enhanced in the gut tissue, with a powerful, suppressive, unchanging phenotype. In a mice model, the deficiency of these cells demonstrated that they are essential to preserving tolerance to microbiota. Microbiota-induced Treg cells prevent colitis [29].

**Figure 1:**
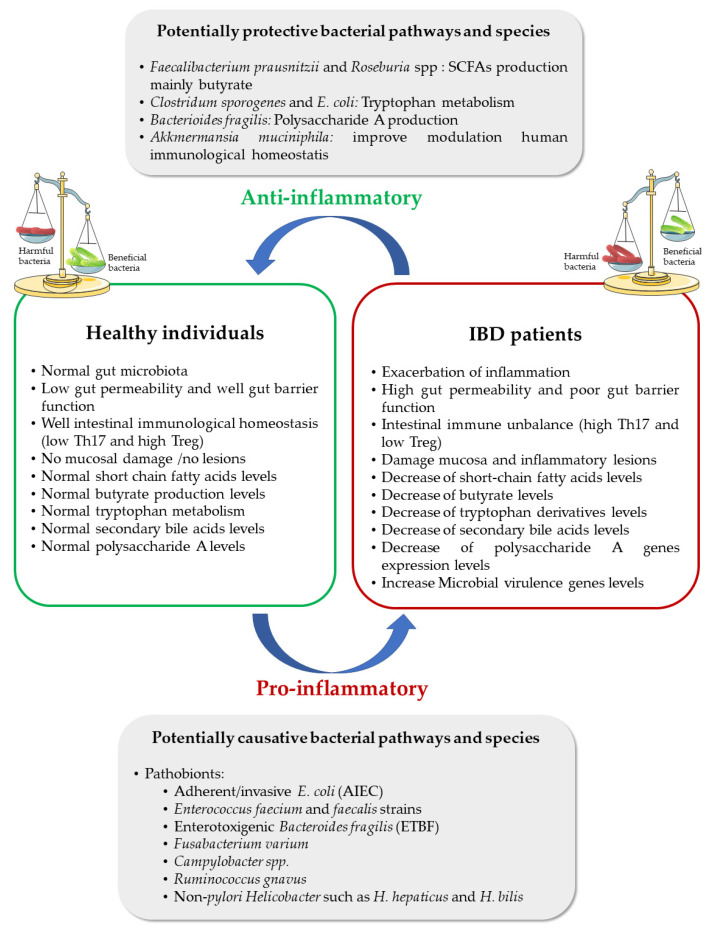
Schematic representation of potentially protective and causative bacteria in IBD, along with the main mechanisms associated with their bioactivity in the bowel. Protective bacteria, such as probiotics, have an anti-inflammatory effect in biological models, whereas causative bacteria for IBD, such as pathobionts, have a pro-inflammatory effect. Adapted from [30,36].

Several studies have recognized variances in gut microbiota biodiversity and species richness between healthy individuals and IBD patients, particularly in the phylum of Firmicutes and Bacteroides. Health gut microbiota are composed of Firmicutes < Bacteroidetes < Proteobacteria < Actinobacteria. IBD patients have fewer bacteria with protective proprieties, such as *Bifidobacterium* spp., *Bacteroides* or *Faecailbacterium prausnitzii* and *Roseburia* spp., and more with pro-inflammatory activities, mainly *Veillomellaceae, Pasteurellacae, Escherichia coli* (*E. coli*, adherent/invasive) and *Fusobacteriaceae* (Figure 1) [30,31,32]. Dysbiosis in UC showed a higher amount of Actinobacteria and Proteobacteria and a lower amount of Bacteroides (Firmicutes < Proteobacteria < Bacteroidetes < Actinobacteria) [33], whereas dysbiosis in CD has shown an even lower amount of Firmicutes phylum than in healthy individuals [34], such as *F. prausnitzii*, which is often proportionally decreased in the patients’ stool [22,35].

Although no pathogenic bacterium has been recognized as causative factor of IBD, many studies have reported the potential implication of microbial pathogens, such as *Mycobacterium avium paratuberculosis* [37], *Clostridium difficile* [38], *Campylobacter* and *Helicobacter* spp. [39,40], and functionally changed commensal bacteria, namely, *Bacteroides fragilis* and *E. coli* [41,42].

A dysbiotic condition can be associated with prior use of antibiotics, and this can lead to the progression of IBD, at early stages. Changes in gut microbiota are the cause or consequence of the inflammation needed for an appropriate diagnosis, selection of therapy, and strategy to monitor response to treatment. Some studies show that dysbiosis may be a cause of IBD and T-cell-mediated chronic colitis [29,43]. The disequilibrium between anaerobe species (obligate and elective) and the oxidative stress induced by gut microbiota can be correlated [27]. The perpetuated inflammation of the intestinal tissue then begins, and enhances the release of haemoglobin, thereby transporting reactive oxygen species and oxygen into the inner intestinal wall, creating a microenvironment that is unfavourable to extremely oxygen-sensitive bacteria. This results in a reduction in obligate anaerobes, mainly *F. prausnitzii*, and causes a severe decrease in butyrate-producing obligate anaerobes and an increase in inflammation by thedepletion of anti-inflammatory proprieties of butyrate [27,44]. The IECs are fuelled by butyrate, which is needed to protect the gut epithelial barrier from becoming vulnerable to potential pathogens. Machiels et al. emphasized that a lower abundance of *F. prausnitzii* and *Roseburia hominis* exists in UC patients than in healthy individuals, which shows a reduction in the butyrate-producing bacteria of this Firmicutes phylum [32]. Depending on disease severity, gut microbial metabolites could encourage the pathogenic Th2 production by human dendritic cells, to the detriment of tolerogenic Th1 cells. Intestinal microbes of IBD patients also have decreased tryptophan-derived indole derivatives, which are known to induce production of the pro-inflammatory IL-22 owing to a gut imbalance [45]. Bergmann et al. showed that the uptake of tryptophan-metabolizing *Lactobacillus* species re-established IL-22 production within the gut and relieved its associated inflammatory status by producing IL-1β in the injured bowel and controlling the following IL-22 increase due to the activity of group 3 innate lymphoid cells. The potential of *Lactobacillus* strains to diminish colitis suggests that their gut metabolites are involved in IBD [45,46]. Sokol et al. also reported that *F. prausnitzii* can secrete metabolites that are able to block IL-8 production and NF-κB activation, as well as induce the production of IL-10 and limit the production of pro-inflammatory cytokines, mainly IFN-γ and IL-12 [47].

*B. fragilis* is a Gram-negative bacterium of the regular human gut microbiota, which has both pro- and anti-inflammatory effects. *B. fragilis* produces the polysaccharide A (PSA) that, in turn, mediate the upregulation and secretion of IFN-γ, TNF-α and IL-6, managing the development of adaptative T helper cells CD4 [41]. *Akkermansia muciniphila* uses mucin as a substrate to produce mucolytic enzymes that cleave mucin’s sulphated terminal chains, thus influencing the gut microbial balance. *A. muciniphila* also reinforces the gut barrier function by promoting the interaction between its outer membrane protein Amuc_1100 and Toll-like receptor 2 [48,49]. Lower levels of *A. muciniphila* correlate with a higher inflammatory status [48]. However, some studies reported that around 40% of ileal colitis disease patients have an overgrowth of adherent/invasive *E. coli* (AIEC) compared to healthy individuals [26,50,51]. These promote IBD, as their genome can adapt to a specific and susceptible host [52]. AIEC can evade the immune system of the host and link to IECs while overpowering autophagy in IBD patients. This bacterium traverses the intestinal wall into the lamina propria, and is then overwhelmed by macrophages that secrete high levels of TNF-α and pursue Th1/Th17 differentiation without host cell death, gut inflammation and AIEC overgrowth [45,53,54]. Several virulence factors confer abilities to adhere, invade, survive, and replicate within host cells to AIEC. Leccese et al. verified that the probiotics *Lactobacillus* and *Bifidobacterium* reduce AIEC virulence mechanisms, interfering with the IL23/Th17 inflammatory response, and controlling their adherence, invasive and survival skills inside IECs, dendritic cells and macrophages collected from IBD patients [53]. Other studies reported on the association between *Mycobacterium avium* subspecies *paratuberculosis* (MAP) infection and CD, suggesting MAP as a potential cause of the disease. However, a lower incidence of MAP infection among CD has also been found [55,56]. Persistent infection and the subsequent proliferation of MAP within human-monocyte-derived dendritic cells could provide a route for the dissemination of MAP in the gut, changing immune responses and encouraging destructive T cell responses, following gut injury in CD patients. MAP infection and proliferation led to a lag in the cells’ maturation, an increase in anti-inflammatory cytokine CD103 and persistent infection [37]. *Clostridium difficile* infections have also been appointed as causative agents of IBD [57]. Shoaei et al. studied the presence of *C. difficile* in faecal samples in UC patients, revealing that all patients with *C. difficile* infection exhibited moderate-to-severe IBD, correlated with exposure to different antimicrobial and anti-inflammatory agents [58].

Fungi, on another hand, represent <0.1% of the total amount of microbial species living in the intestine. In healthy people, *Candida*, *Saccharomyces*, and *Cladosporium* are the most predominant genera; however, in IBD, the gut microbiota reveal an elevated presence of fungi such as *Basidiomycota*, *Ascomycota* and *Candida albicans* [59]. Bacterial biodiversity decreases in CD and UC, while fungal biodiversity only decreases in UC [60]. CD patients exhibit a higher fungal burden over the inflammatory process, changing the ileal physiology in the terminal ileum, which impairs the inhibitory effect of antimicrobial peptides on bacteria and bile acid reabsorption. This explains why an enhanced load of *Candida* species is observed in CD patients, in parallel with disease severity [61]. Sokol et al. showed that *Saccharomyces cerevisiae* (*S. cerevisiae*) is a major component of the healthy fungal microbiota, with a reduction that is independently associated with IBD. *S. cerevisiae* is able to reduce the colitis induced by AIEC, opening a new approach to use fungi as a new therapeutic strategy due to its regulatory effects on the host, such as an anti-inflammatory IL-10 production [60]. Changes in IBD patients’ microbiota include an enhanced fungi/bacteria diversity ratio and high abundance of *C. albicans*, showing an overgrowth of fungi over inflammation. Specific fungi/bacteria interactions may even be important in IBD. Hoarau et al. identified that the abundance of *Candida tropicalis* was high in CD samples and positively correlated with levels of anti-*S. cerevisiae* antibodies (ASCA). Positive interkingdom correlations between *C. tropicalis*, *E. coli*,and *Serratia marcescens* in CD patients were validated using in vitro biofilms, suggesting that these organisms interact in the gut [62].

## 3. IBD Symptoms and Treatment Options

Common genetic abnormalities, immune dysfunction mechanisms, systemic inflammation, dysregulation of gut microbiota, and wide therapeutic overlaps support the hypothesis of a common pathogenesis between CD and UC; the differences occur in terms of the site and nature of the inflamed lesions [7,8,10].

CD provokes segmental, asymmetrical, and transmural lesions, affecting all the digestive tract, with 30% of the cases being installed within the distal parts of the small intestine, while UC only affects the superficial mucosa of the colon and occurs continuously, and circumferentially, from the anus [7,8,63,64]. Endoscopy in CD patients typically reveals a discontinuous distribution of longitudinal apthoid ulcers along the mesenteric aspect, wherein intestinal blood and lymphatic vessels assemble. In mild forms of the disease, superficial ulcers are formed, whereas deep serpiginous ulcers with modular oedematous mucosa are developed in moderate-to-severe cases, producing the so-called cobblestone appearance [7,65]. The non-necrotizing epithelioid and intralymphatic cell granulomas emerge in the focal points, juxta-positioned with endothelial lesions, with the damage suggesting an infectious setting, lymphatic endothelial cell death and granulomatous response, in and around the lymphatic, submucosal, muscular and subserosal layers [7,13,64,65]. This process is specific to CD and is not observed in other chronic forms of enteritis [64]. The extent of these lesions closely correlates with transmural inflammation, fibrosis, muscularization, and stricture formation, and is considered an active participant in intestinal inflammation, in a pathogenic process supporting the release of pro-adipokines and local amplification of inflammation in response to recurrent intestinal ulcerations, which are ineluctably accompanied by bacterial translocation [13,64]. Half of the patients may experience peri-anal complications such as strictures, as well as abscesses and fistulas, within the first decade after diagnosis [5,10].

On the other hand, UC lesions include clearly defined inflamed mucosa and sub-mucosa of the colon and rectum lining, instigating ulcer development [19]. The crypt architecture appears distorted, crypt length is shortened, more lymphocytes and plasma cells appear in the lamina propria, mucin is depleted, and Paneth cells transdifferentiate into other cell types. Severe UC may also comprise toxic megacolon, with colonic dilation visible through abdominal imaging. This is a surgical emergency, given the risk of potential perforation and sepsis [8,66]. Although normally shielded by a thick mucin coating that separates antigens and gut immune cells, mucosal injuries begin with the disruption of the epithelium, the peripheral mucosal layer, and exert antimicrobial activity. As mucin synthesis and secretion is diminished, mucosal internalization of luminal pathogens, antigen uptake and potential stimulation of the gut’s handicapped and intolerant immune system increases [19]. UC can also evolve into dysmotility, anorectal incontinence, pseudopolyposis, bridging fibrosis, and strictures, either from disease progression or postoperative complications [67].

IBD patients often complain of abdominal discomfort and pain, urgency, increased bowel movements, mucus discharge, diarrhoea, incontinence, bloody stool, anaemia, weight loss, fatigue, fever, and/or (in some cases) anal lesions, even though clinical symptoms do not always mirror the underlining inflammatory status and disease severity [3,10,63,68]. Intra- and extraintestinal complications may also occur, involving dermatological, vascular, arthropathy, inflammatory, ocular, respiratory and hepatobiliary, among other disorders, such as metabolic dysfunctions [5,7,8]. Malignancies lead to the second highest IBD mortality rates, after cardiovascular pathologies. These patients have an increased chance of developing carcinoma of the GIT, namely small bowel adenocarcinoma and colorectal carcinoma. The likelihood of IBD progression into colorectal cancer (CRC) is 2.4-fold higher in UC patients than in people devoid of this disorder [63]. The addition of primary sclerosingcholangitis to the equation enhances CRC probability by five times. Hence, close surveillance is recommended immediately after disease diagnosis [8,69]. Extraintestinal malignancies, such as skin, hepatobiliary and hematologic cancers, have also been diagnosed [7,8,69,70]. IBD is also frequently connected with secondary health problems, which are physiopathologically detached from the primary illness, the selected treatment courses and their long-term effects [71]. The early detection of comorbidities is essential in IBD because these conditions can modify disease prognosis, disease activity, and influence pharmacological therapeutic approaches (e.g., drug interactions causing collateral damage, weakened drug bioactivity, and contraindications) [72]. Infections affect 39.8%, 13.2% and 12.9% of IBD patients with pneumonia, sepsis, or candidiasis, respectively [73]. Old-age susceptibility, anti-TNF-α or immunomodulatory therapy, polypharmacy and the presence of other comorbidities, such as diabetes (19.2%), cancer (16.5%), anaemia (16.3%) and chronic obstructive pulmonary disease (COPD) (11.4%), are described as the main factors of this high risk of infection, present in IBD patients [36,74]. Table S1 identifies the main IBD-associated comorbidities.

Current therapeutic interventions in IBD mainly target active inflammatory signals in an attempt to hinder the evolution of the cascade of pro-inflammatory and destructive signals within the intestine’s microenvironment, impeding the development of irreversible bowel injury and the resulting disability [10,13,67]. Medical management is adjusted to disease subtype, severity, location, behaviour, age at diagnosis, lesion extension, presence of intra- or extraintestinal manifestations, or malignancies [7,8,13].

The current therapeutic approach includes 5-aminosalicylates (5-ASA), corticosteroids and immunosuppressants, indicated for mild-to-moderate IBD. More than 90% of UC patients are treated with oral or rectal administration of 5-ASA, shortly after disease diagnosis, particularly mesalamine [7,8,75,76]. If insufficient, oral or intravenous corticosteroids [8,77] may induce remission in mild-to-moderately active UC and CD and are used as a rescue therapy in disease flares [78,79]. Preference is given to the use of oral corticosteroids esuch asprednisone and budesonide [77,80]. Immunosuppressants such as thiopurines are used to maintain remission of UC and CD, after surgery in CD, and as a maintenance strategy after rescue therapy [5,75,79]. Methotrexate presents advantages over thiopurines, such as only requiring a single dose per week, and possessing higher adherence rates and faster onset of action [79], and is, therefore, increasingly used to treat CD [7,8]. 

Upon failure of these drugs due to steroid dependency or unresponsiveness, conventional step-up pharmacological intervention strategy considers targeted biologic therapy as the standard of care [67,81], either used alone or as a co-adjuvant therapy [82]. These targeted therapies (via monoclonal antibodies or small molecules) have been effective in achieving remission and complete mucosal healing in a significant portion of moderate-to-severe cases of CD and UC [83,84], despite their only being effective in a proportion of patients [81]. Some clinicians additionally claim that an early introduction of biologics can, in some cases, further benefit the patients, compared to the traditional treatment course [82]. Anti-TNF-α drugs, specifically adalimumab, infliximab, certolizumab pegol (CD-exclusive) and golimumab (UC-exclusive), are used to treat IBD [8,83,85]. These are widely known monoclonal antibodies which work against TNF-α [77,86,87] and are capable of inducing remission in nearly 50% of patients [86]. Following anti-TNF agents, given their non-negligible rate of loss of response, contraindications, adverse events, and intolerance [81,87], biological therapy can resort to anti-integrins, especially vedolizumab and natalizumab. Integrins are transmembrane receptors that act upon various leukocyte signalling pathways, including cell adhesion, proliferation, and migration [88]. These drugs comprise monoclonal antibodies targeting α4β7 integrins (proteins responsible for the regular migration of leukocytes, preventing leukocyte migration to the gut) and/or α4β1 integrins (with known roles in leukocyte adhesion, spreading, and motility, as well as T cell recruitment to intestinal and non-intestinal inflamed tissues) can be used [75,77,88,89]. Moreover, a recently approved anti-interleukin agent, namely, ustekinumab, may be directed towards the p40 subunit of pro-inflammatory interleukin-12 (IL-12) and interleukin-23 (IL-23) of CD and UC patients [86,90]. The induction dose is administered intravenously, and the following maintenance doses are subcutaneous, which is an advantage for the patient [86]. The inhibition of activated T cells using small molecules that inhibit the enzyme calcineurin–cyclosporine and tacrolimus has also been useful to UC patients who are unresponsive to thiopurines or anti-TNF as an induction therapy in the prevention of UC-induced colectomy, or combined with vedolizumab to stabilize the disease. It may also be used in cases of drug contraindications and rescue therapy in IBD [79,91]. In patients in which conventional and/or biological therapies have not worked, Janus kinase (JAK) inhibitors have been considered as an alternative for UC management. Tofacitinib, with a small-molecule JAK inhibitor, was recently licensed for oral treatment of moderate-to-severe active UC [77,92]. It inhibits all JAKs (preferably JAK1 and JAK3, members of the tyrosine kinase family, which are involved in cytokine signalling), affecting cytokine production and enabling immunomodulation in IBD [84,87]. The simultaneous inhibition of multiple cytokines leads to a lower risk of immunogenicity, which is an advantage compared to the aforementioned therapies, which are associated with monoclonal antibodies [87]. A large number of small-molecule JAK inhibitors are currently under investigation [93], constituting, in parallel with sphingosine-1-phosphate receptor 1 (S1PR1) agonists (e.g., ozanimod and etrasimod), new and attractive treatment tools for parenteral administration [5,94]. Modulation of S1PR activity is needed for lymphocyte blood circulation, additionally enabling lymphocyte entrapment in lymphatic structures [95]. Antisense oligonucleotides (AGO), short nucleotide sequences, inhibit RNA or DNA transcription or translation through complementary base pairing. Alicaforsen specifically binds to ICAM-1 mRNA, thereby reducing the mRNA levels and inhibiting ICAM-1 translation. ICAM-1 is a glycoprotein expressed on the surface of intestinal epithelial cells and vascular endothelial cells, with promising results in terms of UC management, including safety and potentially long-lasting effects. Cobitolimod, an AGO-simulating bacterial DNA used to activate Toll-like receptors 9, is another relevant example [96]. To date, the clinically approved targeted therapies (i.e., monoclonal antibodies and small molecules) constitute the standard of care for moderate-to-severe IBD; however, they are only effective in a portion of the patients [81].

Surgical resection is necessary to remove diseased tissue, and is frequently reserved for severe IBD patients with complications or an intractable disease. Surgical resection provides symptom relief and allows patients to regain their previous quality of life [7,8,67]. Typically, in the first decade as a CD patient, half of the cases become complicated through the formation of strictures or fistulae, with nearly 30% of patients facing multiple surgeries. About 14% of severe CD cases involving complicated rectal disease need a permanently functional stoma [5,63]. Even when less frequent, UC can also cause multiple complications. Nearly 10% of UC patients who are also in their first decade as IBD patients will require colon resection to attempt to control these complications, even though 30% of patients are estimated to present a postoperative complication [67]. However, recent advances suggest that surgical interventions could instead be used as a first-line therapy if combined with biologics (e.g., ileocolic resection for limited disease) and using minimally invasive approaches [97]. Mesenchymal stem cell (MSC) therapy has also been approved in the treatment of active CD fistula, with autologous MSC transplantation becoming increasingly recognized as an upcoming last-resort treatment in severe and unresponsive CD, particularly following clarification of the reservations around the long-term effects and cost-effectiveness of this therapeutic approach [82,95]. The use of antibiotics is mainly reserved for CD complications, although they can also be used to counteract bacterial overgrowth and the occurrence of infection at the wound site of a surgical procedure. In addition, they can be used to maintain remission or to treat bolsite. The most antibiotics that are most frequently employed in CD care are metronidazole, ciprofloxacin, ornidazole, and rifaximin [7,76,98,99]. Metronidazole and ciprofloxacin are effective for anal lesions. In addition to ornidazole, these biomolecules may delay postoperative recurrence [23,98]. 

Microbiota are also being used to treat IBD. Phage-targeted therapy is gaining momentum in the fight against infectious diseases. Typically, phages lyse a subgroup of strains inside a bacterial species [100]. As an example, bacteriophages that target the AIEC strains seem to be a new, promising therapy against IBD, as Galtier et al. demonstrated in transgenic mice [101]. Probiotics, prebiotics or postbiotics have been described as replenishing bacteria, as well as their substrates, which are able to reduce the inflammatory status of the GIT. Probiotics comprise live microorganisms that exert beneficial effects on the host when administrated in large enough quantities, while a prebiotic is a substrate that is selectively used by probiotics for the same purpose. Postbiotics are biomolecules that are produced by a probiotic [100]. As previously mentioned, probiotics can alter the mucosal immune response and help Th1 cell differentiation through Toll-like receptors; this enhances intestinal barrier function and bacterial diversity, and reduces harmful bacteria overgrowth. Probiotics such as *Lactobacillus* spp., *Bifidobacterium* spp., and *Streptococcus salivarius* have been known to enhance the mucosal regulatory T cell number and reduce pro-inflammatory IL-1β, which could promote tolerance instead of pathogenicity [102]. *A. muciniphila* can be administrated orally and improve DSS-induced colitis in fourteen days, as well as enhancing the barrier function [103]. Wang et al. showed that *A. muciniphila* is able to reduce pro-inflammatory macrophages and CD8^+^ T cells in the colonic tissue [104]. The use of prebiotics such as inulin is another strategy to modulate the gut microbiota. Inulin has been described as an inducer of SCFAs producer growth, such as *Lactobacillus* and *Bifidobacterium* spp., and, consequently, as an inhibitor of mucosal lesions and inflammation [105]. Using a different stratety, faecal microbiota transplantation (FMT) can effectively rearrange UC gut microbiota to a healthier state. Faecal microbiota are transferred from a healthy donor into a patient’s GIT, with already proven value in treating persistent *C. difficile* infection [100]. FMT aims to correct the dysbiosis associated with IBD and restore gut microbial homeostasis [100,102]. The changeable triumph of the FMT therapeutic approach is likely associated with the bacterial species present in the donor stool and gut microbiota alterations, leading to a move away from IBD indications. Patients in remission after FMT were seen to display higher levels of microbial diversity and an upgrade in *Eubacterium hallii* and *Roseburia inulivorans* in faecal and colonic tissue samples compared to non-treated IBD patients. An enhancement of SCFAs biosynthesis and secondary bile acid secretion is also expected [102]. However, it is important to highlight that the presence of gut fungal and viral community in donor stool can compromise and decrease FMT efficacy in IBD treatment [100,106]. Table 1 summarizes the potential of microbiota-inspired approaches in the prevention, diagnosis and treatment of IBD [102,107,108].

If ineffectively treated, IBD can lead to an unceasing bowel injury with enhanced risk of hospitalization, surgical procedures, cancer, and comorbidities. Hence, numerous treatments have been defined to treat IBD in all its dimensions, with each option showing benefits to, and side effects for, the patient. Traditional treatment modalities are generally used, but all clinically approved treatments are presently incapable of reversing recurrent bowel damage once it has appeared, thus halting the natural progression of the disease that tends to result, in the long-term, in end-organ bowel injury [63]. IBD can only be managed, not cured [3,67]. Emergent treatment strategies such as treat-to-target (T2T), tight control, treat-to-clear, the use of bispecific antibodies and dual-targeted therapy, seem promising [63,81,106]. The latter involves the combination of two targeted therapies and may be useful for patients with concomitant IBD and extraintestinal manifestations, or in patients with refractory IBD who lack valid alternatives, while having an overall acceptable safety profile. The clinical outcomes after vedolizumab and calcineurin inhibitor action are encouraging [81]. A head-to-head comparison of biological drugs for IBD treatment is ongoing, with the available data already supporting clinicians in the correct choice between different biologicals for each patient [109]. However, drug delivery to the target site is challenging, and has considerable side effects [110]. Thus, their integration into suitable carriers, which are able to protect the drug from biodegradation, transport it into the target site, enable a controlled release and avoid off-target action, can enhance drug exposure to the intended areas and potentiate strong and sustained bioactivity [111,112,113] using lower doses than if they were used in the free form [114,115].

Overall, the achievement of a deep and durable remission is the main therapeutic target when treating IBD, in which clinical, biochemical remission and mucosal healing is attained and maintained [1,63,82,116]. 

## 4. Benefits of a Nanomedicine-Based Therapy for IBD

Nanomedicine approaches allow for the development of therapeutic formulations designed to enhance drug uptake (absorption) into diseased tissues in the colon or other regions of the GIT [117], thus contributing to localized therapy [118]. Nanoparticles (NPs) can access the intestinal mucosa for site-specific drug delivery. Different compositions, sizes, surface charges and coatings have been shown to successfully reach the inflamed intestinal tissues [119]. The adhesion of NPs to the mucus layer results in a prolonged intestinal transit time. Stimuli-responsive delivery systems also display improved drug delivery, directed at the diseased tissues [120]. 

The diseased state of the intestinal tissues alters the local physiology and, consequently, the transit time, mobility, composition, pH and gut microbiota. NPs have shown promising results in overcoming these physical alterations in IBD, strategically augmenting delivered drug amounts at the target site for improved action. In the next section, examples of biodegradable polymeric NPs, developed for the treatment of IBD, will be discussed. Among the various carriers proposed for drug delivery, which have been extensively reviewed elsewhere [121,122,123,124,125], polymeric NPs have been studied for several decades. Polymeric NPs present benefits over other types of nanosystems in terms of drug delivery, namely, a growing choice of biodegradable and biocompatible polymers, easy production methods, higher loading efficiencies, higher stability in physiological environments and increased drug bioavailability [126]. Aliphatic polyesters are the most common polymers employed in NP production, including polylactide, polyglycolide, and co-polymers. They are approved by the Food and Drug Administration (USA) and have well-characterized biocompatibility and (bio)degradability properties. Nanomedicine-based therapy for IBD is currently focused on the exploitation of the modulation of inflammatory environment. 

### 4.1. 5-Aminosalicylic Acid (5-ASA)

5-ASA treats active UC following oral or rectal administration. The mechanistic pathways induced by 5-ASA are still uncertain; however, this drug exhibits anti-inflammatory, immunosuppressive and antioxidant activities. However, very high concentrations of the drug are required to render the treatments effective for IBD. Furthermore, due to their quick and extensive absorption in the upper GIT, poor drug absorption rates are normally found in the colon, diminishing the efficacy of this treatment [127]. Hence, a targeted 5-ASA release to the colonic region may improve therapy outcomes.

In this context, Malviya et al. [128] designed and developed a self-assembled aloe vera acemannan polysaccharide and acrylonitrile NPs, with 50 nm average diameter. The study encompassed an extensive characterization of the formulation and in vitro studies. 5-ASA-loaded optimized formulations have been examined in vitro in different buffered solutions at 37 °C, while comparing acidic (pH 1.2) and neutral (pH 7.4) conditions, to show a colon-specific sustained drug release lasting for up to 48 h. This delayed release of 5-ASA provides time for drug release in the colon rather than in the upper GIT. The network structure of these NPs regulated their swelling ratio, allowing for a controlled release of the entrapped drug at the target pH of the GIT. Further in vivo studies may evaluate the real potential of this formulation.

Following a pH-sensitive swelling approach, Singh et al. [129] developed carboxymethyl cellulose-rosin gum hybrid NPs with a 267 nm average diameter. In vitro colon-specific 5-ASA release from the formulation was examined in simulated gastric and intestinal fluids. Very low quantities of the drug were detected in the gastric medium for the first 2 h. Conversely, in the intestinal fluid, 72% of the drug was progressively released over 12 h. Again, the delayed release from these NPs may increase the bioavailability of the drug in the colon. However, this work focused on the optimization of the formulation and lacked in vivo testing. 

pH responsiveness, along with a mucoadhesiveness strategy was followed, by Cesar and collaborators [127]. They developed a polymeric prodrug by linking chondroitin sulfate with 5-ASA via carbodiimide chemistry as a ligand strategy. The in vitro release showed that around 40% of the conjugated drug was released in basic conditions (pH 9) for a period of up to 50 h. The loaded NPs were cytocompatible towards the human monocytic cell line, and a murine in vivo biodistribution study displayed the conjugate in the lower GIT for up to 8 h, together with a null presence in the upper GIT. These data corroborated the colonic mucoadhesiveness of the tested formulation, as desired. 

Following a mucoadhesive strategy, Bahadori et al. [130] engineered core-shell NPs, containing anionic sodium-alginate-coated and quaternized inulin and encapsulating 5-ASA. The core-shell structured formulation relied on a bio-adhesion trait attributable to the existence of a high mucoadhesive inulin shell, a feature that increased NP stability in the upper GIT and reduced NP degradation in intestinal conditions. Two formulations, with ≈84–100 nm and ≈156–198 nm of core and shell diameter, respectively, showed a slow-release profile of 5-ASA. The authors claimed that the particle size and shape are appropriate for oral administration, and that the inulin coating, by itself, could additionally assist IBD treatment as a prebiotic. However, biocompatibility assays and in vivo efficacy assays were lacking.

In a different approach, Nalinbenjapun et al. [131], developed a chitosan-5-ASA azo-conjugate for targeted delivery to the colon. The freeze-dried chitosan-5-ASA azo-conjugates consisted of microparticle aggregates of around 0.5 μm in diameter, which were soluble in basic media (pH 14) and insoluble in simulated GI fluids. However, the authors reduced particle size to the 100 nm range to increase the particle surface area and azo bond exposure to bacterial enzymes, such as azoreductase, for enhanced 5-ASA bioactivity. Anyhow, the developed formulation was determined to be stable and only released around 25% of 5-ASA in simulated gastric, intestinal and colon fluids for 24 h at 37 °C.

Another problem associated with IBD is the high expression levels of myeloperoxidase (MPO), which, through a cascade of events, may cause damage at the site of inflammation [132]. In this sense, Iwao et al. [133], developed 5-ASA-loaded human serum albumin (HSA) NPs, to take advantage of the communication between MPO and HSA. The formulation presented 190 nm as averaged particle diameter, a polydispersity index of 0.35 and zeta potential of ≈−11 mV. The specific affinity between NPs and MPO was explored in the imaging of colonic tissue sections, after being collected from the used DSS-induced colitis mice model, demonstrating that HSA NPs and MPO were co-localized in the colonic tissue. Mild inflammatory damage could also be perceived, but this still suggests mucosal repair.

### 4.2. Corticosteroids

Corticosteroids, together with 5-ASA, are the cornerstones of IBD therapy, and are mainly used to induce remission. Although highly effective, therapy with corticosteroids is hampered by their serious side effects related to off-target interactions. Among the corticosteroids, budesonide (BUD) is the first choice in the treatment of IBD, due to its potent local anti-inflammatory activity and fewer side effects than other corticosteroids. Nevertheless, it still has deleterious side effects and poor aqueous solubility, justifying the encapsulation. 

In this context, Ali et al. [134] created BUD-loaded pH-responsive polymeric NPs of 240 nm in diameter, composed of a PLGA core and an enteric coating layer with a methacrylate copolymer (Eudragit^®^ S100). Following oral administration, the purpose of this work was to limit early BUD release in acidic gastric conditions so that a slow and sustained drug release could occur in the distal part of the GIT. In vitro studies showed that the pH-sensitive coating vetoed early BUD release at an acidic pH. The efficacy of the polymeric formulations was evaluated in acute and chronic mouse models of colitis. Eudragit S100-coated PLGA NPs were more effective in decreasing IBD symptoms compared to uncoated NPs and BUD-free solution. Additionally, fluorescence imaging analysis confirmed that the optimized NPs were capable of enhancing retention in the colon. With the same purpose of preventing early BUD release in the upper GIT, Sinhmar et al. [135] used Eudragit^®^ S100 to incorporate mannosylated nanostructured lipid carriers, which enabled the active targeting of BUD to the inflamed tissues. The active targeting relies on the fact that mannose receptors [175-kDa transmembrane protein of the C-type lectin family (CLR)] are exclusively overexpressed on the surface of pro-inflammatory macrophages. The in vitro drug release studies revealed that the coating led to a lag time release of 5 h (transit time needed to arrive at the colonic tissue). Moreover, in vivo screening, performed in an oxazolone-induced colitis rat model, showed that the formulation significantly reduced the clinical signs of disease, colonic MPO action and pro-inflammatory cytokine panel.

More recently, Qelliny and co-workers [136] developed pH-sensitive BUD-loaded Eudragit^®^ S100/Capryol 90 nanocapsules to solve the insufficient BUD amounts in diseased regions by preventing its release in non-inflamed GIT regions. The nanocapsules, optimized by a full 31 × 21 factorial design, presented a mean size of 171 nm, polydispersity around 0.127 and a negative zeta potential of about −37.6 mV. They presented a low burst release of 10% for the first 2 h and a higher rapid cumulative release of 72% after 6 h. The in vivo efficacy was studied using a rat colitis model. In parallel with free BUD suspension, the produced formulation was capable of improving disease activity score, macroscopic view, blood glucose levels, and histopathological signals. 

In a different approach, Sun et al. [137] developed redox-sensitive NPs built from amphiphilic inulin and 4-aminothiophenol (ATP) grafted onto a carboxymethyl inulin (CMI), carrying BUD. The particles’ average size was 210 nm, while the zeta potential was around −14 mV. The in vitro BUD release studies in glutathione (GSH)-free simulated gastric fluid showed a low BUD release rate (about 45%), whereas a high release rate (near 80%) was obtained in media with 20 mM GSH, displaying a redox-sensitive capacity. In vivo testing, use of a dextran sulphate sodium (DSS)-induced colitis mice model showed that the NPs accumulated in the inflamed sites, and exerted a greater therapeutic effect than free BUD. This behaviour is attributed to the formation of a covalent linkage between ATP-CMI NPs and mucins, and the redox-triggered release for an augmented drug amount being delivered intracellularly. 

Given that the intestinal mucosal barrier hinders colorectal drug retention and absorption, Date et al. [138] designed BUD nanosuspensions containing muco-inert coatings (Pluronic^®^ F127). They demonstrated that BUD nanosuspension (≈230 nm) enabled increased colorectal tissue BUD collection with minimal systemic counts, compared to a BUD micro-suspension (≈2 μm) prepared with the same stabilizer as the clinical product (polyvinylpyrollidone (PVP)). In vivo experiments, using an acute trinitrobenzenesulfonic acid (TNBS) mouse model of IBD, showed that daily administration of a BUD nanosuspension enema treatment resulted in a significant lessening of IBD symptoms, such as decreased colon weight and histology score, in comparison with untreated controls or free BUD-treated mice. Furthermore, the formulation significantly reduced the presence of M1-macrophages and IL-β-producing CD11b+ cells in the colon.

The combination of dual stimuli-responsive systems was exploited by Li and collaborators [139]. They developed BUD-loaded hyaluronic acid NPs, linked to porous silicon to bind the enzyme-sensitive hydrogel and the pH-responsive hydroxypropyl methylcellulose acetate succinate polymer, assembling a hierarchical (nano-in-nano-in-micro) structure with planned characteristics. The prepared vehicles showed a well-defined spherical shape and homogeneous size distribution. The pH-responsive chains protect BUD and silicon NPs against GIT-induced biodegradation before reaching the target segment of the intestine without early BUD release. Additionally, the anionic hyaluronic acid selectively targets the inflamed intestinal regions, for local BUD release triggered by inflammatory signals. In vivo experiments, in the DDS-induced colitis mice model, a preferred accumulation of the vehicle in the diseased colon was shown. Moreover, the vehicle revealed the lowest disease activity index and pro-inflammatory cytokine levels (IL-6 and IL1-β) when compared with free BUD or delivered by conventional pH-responsive NPs. 

Dexamethasone (DEX) is another corticosteroid that has been encapsulated in polymeric NPs, in an attmpt to improve IBD therapy. Lee et al. [140] developed spherical polymeric nanoconstructs (SPNs), composed of carboxylated poly(lactic-co-glycolic acid) (PLGA) and 1,2-dipalmitoyl-*sn*-glycero-3-phosphocholine (DPPC), encapsulating dexamethasone for the systemic treatment of IBD. The NPs were uniform in size, averaging 162 nm, with a polydispersity of 0.23 and zeta potential of –34 mV. In vitro experiments showed that NP incubation with LPS-stimulated RAW 264.7 cells reduced the inflammatory cytokine profile as quickly as free drug. In a DDS-induced colitis mice model, the formulation counteracted weight loss, pro-inflammatory macrophage presence, expression of pro-inflammatory cytokines, rectal bleeding and histological defects, when compared to the free drug. In a different approach, Wang et al. [141] built self-assembling polyphenols and polymers with a poly(ethylene glycol (PEG)) block encapsulating DEX. The strategy aimed to include polyphenol enzymatic degradation in the UC microenvironment by upregulating esterases, profiting from their strong capacity for radical scavenging, able to consume the produced reactive oxygen species at the inflammatory cluster. The round-shaped NPs, composed of tannic acid and Pluronic F-68, showed the most homogeneous size distribution of all the tested combinations of polyphenols and polymers. These NPs presented a radical-scavenging ability and ROS-triggered release behaviour in simulated intestinal fluid in the presence of esterases. Additionally, an in vivo mice model of colitis revealed enhanced DEX retention rates.

More recently, Mukhtar and colleagues [142] created mannosylated chitosan-based NPs through ionic gelation with tripolyphosphate, and loaded them with DEX to target macrophages for attenuation of early-stage inflammation in IBD. A high dose of DEX may cause abdominal distention and intestinal perforations. Hence, the use of mannose-anchored NPs to transport the drug into pro-inflammatory macrophages revealed, once again, great promise for colon-targeted drug delivery. NPs were further coated with Eudragit^®^ S100 to prevent premature drug release in the stomach. Particles had a diameter of 380 ± 19.8 nm, a zeta potential of −8.25 ± 6.39 mV and an encapsulation efficiency of 78.1 ± 1.17%. An adequate pH-dependent drug release profile was obtained in simulated body fluid at pH 7.4. Cellular studies verified NP biocompatibility and uptake by macrophages. 

### 4.3. Immunomodulators

IBD management has relied on immunomodulators (e.g., azathioprine, methotrexate, and cyclosporine A) from its beginnings. The immunosuppressive properties assure a more extended therapeutic effect than corticosteroids in IBD patients, and are particularly useful in terms of corticosteroid dependency, already showing corticosteroid-unresponsiveness. However, most molecules of this class, when used in IBD treatment, also elicit adverse side effects and present bioavailability issues. One strategy to overcome these drawbacks involves the application of drug delivery systems. In the last 5 years, research has focused on cyclosporine and methotrexate. Wang and colleagues [143] have obtained nanovesicles from grapefruit to deliver methotrexate in intestinal inflamed tissue. A selective uptake by intestinal macrophages was observed, inducing upregulation of heme oxygenase-1 expression, and decreasing IL-1β and TNF-α production. Confocal microscopy revealed the accumulation of nanovesicles in the perinuclear region of the cells and internalization via both micropinocytosis- and clathrin-dependent pathways. However, the detailed molecular mechanisms responsible for interaction with macrophage function have yet to determined. The incorporation of methotrexate in the grapefruit-derived nanovesicles reduced its toxicity and improved the methotrexate therapeutic effects in DSS-induced mouse colitis.

The first immunosuppressant to reach the market was cyclosporine, a peptide isolated from the fungi *Tolypocladium inflatum.* This powerful immunomodulatory agent is widely used in the therapy of inflammatory conditions, including severe IBD stages. The efficient delivery of poorly water-soluble drugs such as cyclosporine has been attempted using different nano-delivery systems. Recently, Naeem and collaborators [144] incorporated cyclosporine in pH-sensitive Eudragit FS30D/PLGA NPs. These smart nanoparticles (ca. 250 nm) prevented the drug burst release at pH 1.2 and 6.8, achieving almost total release at pH 7.4, due to complete NP dissolution. In in vivo distribution studies in murine experimental colitis, treatment with the Eudragit FS30D/PLGA NPs improved weight loss and colon length as well as other inflammatory parameters. Courthion [145] also combined polymers to increase cyclosporine solubility, using a self-assembled NP containing methoxy poly-(ethylene glycol) hexyl substituted poly-(lactic acid) (mPEGhexPLA). These NPs were small (ca. 50 nm), improved cyclosporine retention in inflamed colonic tissues upon rectal administration, and no systemic distribution was detected. The therapeutic efficacy of the NPs was comparable to 5-ASA. Oral administration of cyclosporine loaded on PLGA polymer led to an effective drug-targeted delivery in the inflamed intestinal tissue. Melero and co-workers [146] studied the influence of size using nano- and microparticles loaded with cyclosporine. The Sandimmun Neoral, which is commercially available, was also evaluated using an acute model of murine DSS-induced inflammation model. The NPs improved the local delivery of cyclosporine to the inflamed intestinal tissues, as well as disease indicators at half the dose applied with microparticles, and the commercial formulation, with minimal systemic delivery.

### 4.4. RNA Therapeutic Strategies

A polysaccharide bacteria-degradable hydrogel comprising alginate and chitosan was employed to facilitate the delivery of active agents to the inflamed colonic tissues [147,148,149]. Laroui et al. [148] used this hydrogel as a matrix to deliver polylactide NPs containing CD98 small interfering RNA (CD98siRNA) with colon-homing properties. CD98 expression in the intestine is crucial in the local management of immune responses and homeostasis. The designed CD98 siRNA/polyethyleneimine-polylactic-acid-loaded NPs (ca. 480 nm) were cytocompatible towards intestinal cells. Upon oral administration, the NPs, enclosed in a hydrogel, decreased CD98 expression in colonic cells and reduced colitis parameters in the DSS-induced colitis in a mouse model. Given the crucial role of cytokines and chemokines in IBD progression, Frede and co-workers [150] designed a delivery system for local interference in the signalling pathways. The study evaluated the therapeutic potential of siRNA-loaded calcium phosphate (CaP)/PLGA NPs to modulate gene silencing in epithelial cells. Multi-shell NPs of a CaP core were coated with siRNA directed at mediators of inflammation, such as TNF-α, then encapsulated in PLGA coated with an outer layer of polyethyleneimine. This prevented nanoparticle degradation and conferred them with a cationic surface to enhance cellular uptake. The non-toxic siRNA-loaded calcium phosphate/PLGA NPs were rapidly taken up by MODE-K intestinal epithelial cells; subsequent in vitro gene silencing was observed. Upon intrarectal application of the NPs in a DSS-induced colonic inflammation mouse model, a substantial decrease in the targeted genes (e.g., TNF-α, IP-10) was found in the colonic biopsies and the mesenteric lymph nodes. Amelioration of the intestinal inflammation was achieved with specific management of the inflammatory response using polymeric NPs.

The hydrogel was also used as a matrix for the co-delivery of IL-22 and polymeric NPs containing siRNA [151]. PLGA NPs functionalized with galactose were obtained for the oral delivery of TNF-α siRNA (ca. 260 nm). These smart NPs mediated targeted delivery to macrophages and inhibited the expression of TNF-α. The combination with IL-22, which has mucosal healing properties, yielded an effective therapeutic response against colitis in a mouse model, concerning each factor seperately. The co-delivery of pro-resolving factors could become a promising strategy for oral therapy of inflamed colon. Recently, phosphorothiolated antisense oligodeoxyribonucleotides of TNF-α were embedded in the same chitosan–alginate hydrogel, aiming for intestinal inflammation control [152]. The nanocarrier containing about 83.5% of the oligonucleotide was administered orally in a DSS-induced inflammation mice model. The antisense oligonucleotides were able to attenuate the inflammatory response, providing a new strategy for intestine inflamed targeted therapy. As microRNA 31 expression levels are enhanced in the intestines of IBD patients as well as in colitis-associated neoplasia’s patients, Tian et al. [153] studied the influence of this microRNA and the effect of the delivery of mimics in a colitis mice model. Peptosome NPs were obtained from partially hydrolysed alpha-lactalbumin and loaded on the surface with microRNA 31 mimics. To prevent degradation in the gut, these protein-based NPs were encapsulated in microspheres of oxidized konjac glucomannan, which is enzymatically degraded in the intestine. These were localized in the mice colonic epithelial cells and were able to reduce the inflammatory response, increase body weight and colon length, and cause IEC proliferation.

Macrophage targeting after oral administration of NP dispersion was pursued by Zhang et al. [154] using galactosylated trimethyl chitosan–cysteine (GTC) NPs that are physically crosslinked with tripolyphosphate. Macrophage galactose-type lectin (MGL) is highly expressed at the surface of M1-macrophages, so particles were designed to actively target these cells. CS trimethylation turns the polymeric chains cationic over a larger pH range, increasing NP stability and promoting cell internalization [155,156,157]. Further thiolation with cysteine enhanced their adhesion to mucin glycoproteins through covalent bonds. Mitogen-activated protein kinase kinase kinase kinase-4 siRNA (siMap4k4) was the AGO selected to attempt the suppression of TNF-α production of activated macrophages for UC management. NPs with ≈148 nm particle size and a zeta potential of ≈21 mV were stable in gastrointestinal fluids and were quickly internalized by activated macrophages. In vitro and in vivo testing showed that loaded NPs effectively inhibited TNF-α production, including the efficient distribution of the biomolecule in the ulcerative colon following oral administration. Daily oral administration of loaded NPs significantly improved DSS-induced body weight loss and colon length shortening, and increased MPO activity. 

A different approach was followed by Xu and colleagues [158], in which TNF-α siRNA and DEX sodium phosphate were loaded into a TKPR peptide-functionalized, reversibly crosslinked polymersomes constituted by poly(ethylene glycol)-b-poly(trimethylene carbonate-codithiolane trimethylene carbonate)-b-polyethylenimine (PEG-P(TMC-DTC)-PEI) triblock copolymer. The cationic PEI segments enabled drug encapsulation via electrostatic interactions, while PEG promoted NP furtivity. The pendent dithiolane rings in the P(TMC-DTC) block can form redox-sensitive disulphide bonding, thus conferring enhanced colloidal stability and responsiveness to the NPs. TKPR, a macrophage-targeting peptide, was grafted to PEG terminal moieties for targeting action. These neutral and serum-stable NPs exhibited a spherical and hollow vesicle structure with a diameter of nearly 108–138 nm. About 98% of NPs were efficiently internalized by macrophages. A glutathione-induced drug released was observed, along with efficient gene silencing and anti-inflammatory effect. Intravenous injection of the NPs revealed potent anti-inflammatory action in inflamed colons of UC mice, substantially reducing colonic injury. 

**Figure 2:**
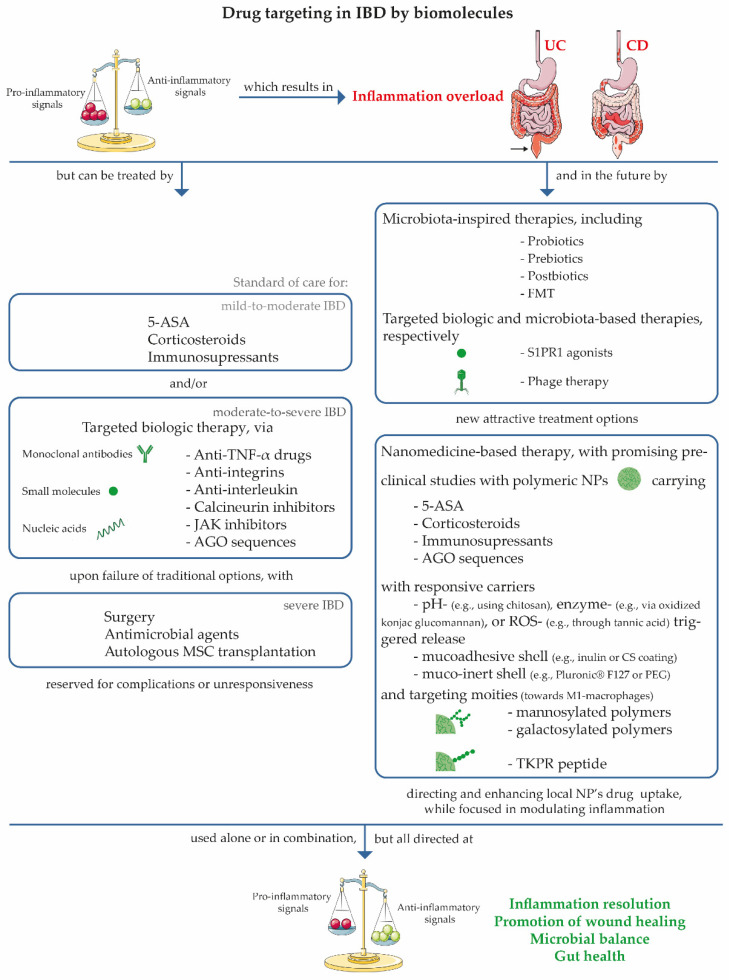
Simplified representation of the main current and potential treatments for IBD, with emphasis on drug targeting in IBD, with elements from Servier Medical Art.

Figure 2 provides an overview of the main current and potential treatments for IBD, indicating the main targeting therapies currently being used in the clinic (monoclonal antibodies, small molecules and nucleic acids), or underway [158,159,160]. Promising results are being obtained with new targeted biomolecules (small molecules and phage therapy) or drugs that are integrated into nanoscaled drug delivery systems, carrying clinically approved drugs, tunable responsiveness, and polymeric chains (carbohydrates and peptides) directed at pro-inflammatory macrophages. 

## 5. Conclusions

IMIDs such as IBDs are characterized by unresolved and aberrant inflammatory conditions, relapsing and remitting disease stages, an obligation for lifelong medication and substantial morbidity [82]. The persistent and intolerant inflammatory state of these diseases creates progressive dysfunction and destruction of healthy tissue, thereby hindering wound healing initiation and endurance. Current therapeutics are ineffective at breaking this detrimental chain [5,7,8]. A large panoply of advanced therapies is accessible at present, but none offer deep remission to most patients [63,160]. Moreover, frequent unresponsiveness to treatment forces patients to test other medications or increase their quantity [160], even though a new IBD treatment paradigm advocates an early top-down treatment approach by resorting to biologic agents in high-risk patients, especially in CD [5,67]. A high amount of any medication may worsen any potential detrimental effects [160]. Thus, the search for new strategies to resolve IBD-associated chronic inflammation, bowel damage and associated conditions is of the utmost importance, with targeted biologic therapies of free drugs currently leading this fight. 

Nanomedicine-based approaches provide innovative and targeted therapeutic options for IBD, as NPs improve delivery to the inflamed intestinal tissue [120,161]. Despite the promising nanoplatforms found in the literature, with enhanced therapeutic efficacy, no delivery system has been approved for clinical use to date [124,162]. The limitations of NPs can be related to the complexity of their design, which hampers the manufacturing process, and their suitability to the intended administration route (oral, rectal or intravenous). In vitro and in vivo murine models have been widely applied to demonstrate the potential application of NPs. Human studies are scarce, but the results are quite different from the ones obtained using animal models. Additionally, the effects of the NPs in human cells are not always explored for the whole GIT, and all the available toxicity and interaction data were obtained in different animal models. To date, there is no definition of a delivery system size range that would enable effective uptake by the cells in the inflamed tissue. There is also no information on the fate of the NPs in the body upon administration, as most of the studies focus on the effectiveness but lack an evaluation of the nanoparticle’s safety. Moving towards the clinical phases of development will most certainly require these safety issues to be addressed, as well as their efficacy as IBD therapeutics.

Manipulating the microbiota via probiotics, prebiotics, or postbiotics, FMT or targeted phage therapy, also have great potential in IBD treatment [82,160,163], as well as in nutritional interventions that can assist in altering enteric flora to cause patient benefit [5,164,165]. Non-absorbable antibiotics, such as rifaximin, along with short-term alternating antibiotics, may also prevent pathogen resistance to antibiotics, thus warranting further study in IBD [5,166]. The goal is to induce a microbial balance.

Collectively, the research has been tireless in proposing new drugs, drug repurposing, and alternative therapeutics, primary or complementary to the existing ones, with all attempting to achieve IBD eradication. Recent IBD treatment options have proven successful for a significant number of patients. However, efforts should be made to create new strategies for adequate patient selection for each treatment, treatment combinations, and/or therapies should be adjusted/reinforced to extend their efficacy to all IBD patients [95].

## Figures and Tables

**Table 1:** Microbiota-inspired therapies to fight IBD.

Predicting treatment		The risk of complications/illness progression. Response to treatment. Personalization of medicine and complementation of the pharmacogenetics approaches currently used in IBD treatment.
Diagnostic tool		Faecal gut microbiome may be a fast and economic strategy to eliminate IBD findings. Help to distinguish IBD subtypes (UC and CD).
Treatment	Drug from Bugs	Bioactive microbiome metabolites such as the production of SCFAs, butyrate-mediated Treg expansion. A targeted approach may allow for the recognition of compounds worthy of fine-tuning to enhance drug selection, efficiency, and improved pharmacokinetic proprieties.
	Bugs as Drug	FMT treatment of pathobiont such as *C. difficile* infection. Probiotics or multiple-bacteria cocktail that offer alternatives for the optimization and standardization of treatments. Specific anti-inflammatory bacteria, such as *F. prausnitzii* or *A. muciniphila*.
	Drug for Bug	Prevent the reduction in microbial concentration in IBD patient’s stool Decrease factors that promote gut dysbiosis: antibiotic treatments enhanced oxidative stress due to intestinal haemorrhage and anaerobic immunoregulatory by-products (nitrate) by the generation of ROS and nitric oxide.
	Decolonization	Phage therapy allows for the species-specific targeting of conserved membrane proteins for phage berthing and load release, culminating in the elimination of the intended microbe through genome modification or control. Identify and remove specific bacterial-induced inflammation using a phage. Antibiotics or other antimicrobial compounds such as peptides and plant extracts.

## Data Availability

Not applicable.

## Supplementary Materials

The following are available online at https://www.mdpi.com/article/10.3390/nano11082035/s1, Table S1: Main IBD-associated comorbidities.
