# Supplementary material for: Drug Targeting of Inflammatory Bowel Diseases by Biomolecules
Source: Nanomaterials (Basel). 2021 Aug 10;11(8):2035. doi: 10.3390/nano11082035 (PMC8401460; doi:10.3390/nano11082035)
Supplement: Supplementary file 1 [file nanomaterials-11-02035-s001.zip › nanomaterials-1294008-supplementary.pdf]

nanomaterials

Review

Drug Targeting of Inflammatory Bowel Diseases by Biomolecules

Joana Costa Antunes [1,*], Catarina Leal Seabra [2], Joana Margarida Domingues [1], Marta Oliveira Teixeira [1], Cláudia Nunes [2], Sofia Antunes Costa-Lima [2], Natália Cândido Homem [1], Salette Reis [2], Maria Teresa Pessoa Amorim [1] and Helena Prado Felgueiras [1]

[1] Centre for Textile Science and Technology (2C2T), Campus de Azurém, University of Minho, 4800-058 Guimarães, Portugal; joana.domingues@2c2t.uminho.pt (J.M.D.); pg35037@alunos.uminho.pt (M.O.T.); natalia.homem@2c2t.uminho.pt (N.C.H.); mtamorim@det.uminho.pt (M.T.P.A.); helena.felgueiras@2c2t.uminho.pt (H.P.F.)

[2] Laboratório Associado para a Química Verde (LAQV), Network of Chemistry and Technology (REQUIMTE), Departamento de Ciências Químicas, Faculdade de Farmácia, Universidade do Porto, 4050-313 Porto, Portugal; cseabra@ff.up.pt (C.L.S.); cdnunes@ff.up.pt (C.N.); slima@ff.up.pt (S.A.C.-L.); shreis@ff.up.pt (S.R.)

* Correspondence: joana.antunes@2c2t.uminho.pt; Tel.: +351-253-510-289

Table S1. Main IBD-associated comorbidities.

Comorbidity	Main appointed cause(s)	Known prevalence	Ref.
Chronic fatigue	Nutritional deficiencies; anaemia; hormonal disturbances; poor sleep quality; psychological aspects; drug side effects	60–80% (active IBD)<br>20–40% (remitted IBD)	[1,2]
Infections	Old-age susceptibility; anti-TNF therapy; IM therapy; polypharmacy; other associated comorbidities	39.8% (pneumonia)<br>13.2% (sepsis)<br>12.9% (candidiasis)	[39,73,74]
Psychological disorders (stress, anxiety; depression)	Increased expression of TNF-α and IL-6 proinflammatory cytokines; high levels of CRP	34.3% (depression)<br>18.6% (anxiety)	[3–7]
Osteoporosis	Steroid treatment; systemic inflammation; calcium and vitamin D deficiencies; malnutrition	14–42%	[8,9]
NAFLD	Increased expression of IL-6 and TNF-α	27.5%	[74,10–12]
PS	Anti-TNF treatment; T cell misbalance induced by $\alpha 4\beta 7$ blockade	2–9%	[13,14]
Thromboembolism	Inflammation; immobility; surgery; steroid therapy; central venous catheters	1–6.7%	[15–17]
AD; vascular dementia; unspecified dementia	-	1.9% (AD)<br>0.7% (vascular dementia)<br>2.9% (unspecified dementia)	[18–20]
Microvascular dysfunction	Diminished vasodilatory capacity to ACh	-	[17,21]

CAD	High levels of TNF-α and IL-6; High levels of CRP, homocysteine and CD40 ligand	-	[71,22]
CVA	Increased thickness of the carotid intimal-media	-	[17,23]
PD	Increased expression TNF-α and IL-1β	-	[24–27]
MS	-	-	[28,29]
Obesity	-	-	[74,30]
Diabetes mellitus	Increased expression of TNF-α	-	[74,31]
COPD	Increased expression of TNF-α, IL-6 and IL-13	-	[32–35]

[1] Abbreviations: Ach: Acetylcholinesterase; AD: Alzheimer's disease; CAD: Coronary artery disease; CNS: Central Nervous System; COPD: Chronic obstructive pulmonary disease; CVA: Cerebrovascular accidents; IM: Immunomodulators; IBD: Inflammatory Bowel Disease; MS: Multiple sclerosis; NAFLD: Non-alcoholic fatty liver disease; PD: Parkinson's disease; PS: Paradoxical Psoriasis; ROS: Reactive oxygen species.

Reference

1. Hindryckx, P.; Laukens, D.; D'Amico, F.; Danese, S. Unmet Needs in IBD: The Case of Fatigue. *Clin. Rev. Allergy Immunol.* **2018**, *55*, 368–378, doi:10.1007/s12016-017-8641-4.
2. Tsai, S.Y.; Chen, H.J.; Lio, C.F.; Kuo, C.F.; Kao, A.C.; Wang, W.S.; Yao, W.C.; Chen, C.; Yang, T.Y. Increased risk of chronic fatigue syndrome in patients with inflammatory bowel disease: A population-based retrospective cohort study. *J. Transl. Med.* **2019**, *17*, 55, doi:10.1186/s12967-019-1797-3.
3. Bernstein, C.N. The Brain-Gut Axis and Stress in Inflammatory Bowel Disease. *Gastroenterol. Clin. N. Am.* **2017**, *46*, 839–846, doi:10.1016/j.gtc.2017.08.006.
4. Kéri, S.; Szabó, C.; Kelemen, O. Expression of Toll-Like Receptors in peripheral blood mononuclear cells and response to cognitive-behavioral therapy in major depressive disorder. *Brain Behav. Immun.* **2014**, *40*, 235–243, doi:10.1016/j.bbi.2014.03.020.
5. Marrie, R.A.; Walld, R.; Bolton, J.M.; Sareen, J.; Walker, J.R.; Patten, S.B.; Singer, A.; Lix, L.M.; Hitchon, C.A.; El-Gabalawy, R.; et al. Rising incidence of psychiatric disorders before diagnosis of immune-mediated inflammatory disease. *Epidemiol. Psychiatr. Sci.* **2019**, *28*, 333–342, doi:10.1017/S2045796017000579.
6. Ohlsson, L.; Gustafsson, A.; Lavant, E.; Suneson, K.; Brundin, L.; Westrin, Å.; Ljunggren, L.; Lindqvist, D. Leaky gut biomarkers in depression and suicidal behavior. *Acta Psychiatr. Scand.* **2019**, *139*, 185–193, doi:10.1111/acps.12978.
7. Sun, Y.; Li, L.; Xie, R.; Wang, B.; Jiang, K.; Cao, H. Stress Triggers Flare of Inflammatory Bowel Disease in Children and Adults. *Front. Pediatr.* **2019**, *7*, 432, doi:10.3389/fped.2019.00432.
8. Farraye, F.A.; Melmed, G.Y.; Lichtenstein, G.R.; Kane, S.V. ACG Clinical Guideline: Preventive Care in Inflammatory Bowel Disease. *Am. J. Gastroenterol.* **2017**, *112*, 241–258, doi:10.1038/ajg.2016.537.
9. Oh, H.; Ryu, K.; Park, B.; Yoon, B. Osteoporosis and Osteoporotic Fractures in Gastrointestinal Disease. *J. Bone Metab.* **2018**, *25*, 213–217, doi:10.11005/jbm.2018.25.4.213.
10. Mundi, M.S.; Velapati, S.; Patel, J.; Kellogg, T.A.; Abu Dayyeh, B.K.; Hurt, R.T. Evolution of NAFLD and Its Management. *Nutr. Clin. Pract.* **2020**, *35*, 72–84, doi:10.1002/ncp.10449.
11. Parthasarathy, G.; Revelo, X.; Malhi, H. Pathogenesis of Nonalcoholic Steatohepatitis: An Overview. *Hepatol. Commun.* **2020**, *4*, 478–492, doi:10.1002/hep4.1479.
12. Zou, Z.Y.; Shen, B.; Fan, J.G. Systematic Review with Meta-analysis: Epidemiology of Nonalcoholic Fatty Liver Disease in Patients with Inflammatory Bowel Disease. *Inflamm. Bowel Dis.* **2019**, *25*, 1764–1772, doi:10.1093/ibd/izz043.
13. Cottone, M.; Sapienza, C.; Macaluso, F.S.; Cannizzaro, M. Psoriasis and Inflammatory Bowel Disease. *Dig. Dis.* **2019**, *37*, 451–457, doi:10.1159/000500116.
14. Vlachos, C.; Gaitanis, G.; Katsanos, K.; Christodoulou, D.; Tsianos, E.; Bassukas, I. Psoriasis and inflammatory bowel disease: Links and risks. *Psoriasis* **2016**, *6*, 73–92, doi:10.2147/PTT.S85194.
15. Morgan, K.; Boktor, M.; Ford, C.; Pham, L.; Morris, J.D.; Jordan, P.A.; Cvek, U.; Trutschl, M.; Alexander, J.S. Venous thromboembolism in IBD: Increased risk for women in CD? *Pathophysiology* **2019**, *26*, 163–168, doi:10.1016/j.pathophys.2019.03.001.
16. Oldenburg, B.; Van Tuyl, B.A.C.; Van Der Griend, R.; Fijnheer, R.; Van Berge Henegouwen, G.P. Risk factors for thromboembolic complications in inflammatory bowel disease: The role of hyperhomocysteinaemia. *Dig. Dis. Sci.* **2005**, *50*, 235–240, doi:10.1007/s10620-005-1588-y.

17. Papa, A.; Danese, S.; Grillo, A.; Gasbarrini, G.; Gasbarrini, A. Review article: Inherited thrombophilia in inflammatory bowel disease. *Am. J. Gastroenterol.* **2003**, *98*, 1247–1251, doi:10.1111/j.1572-0241.2003.07491.x.
18. Antoni, L.; Nuding, S.; Wehkamp, J.; Stange, E.F. Intestinal barrier in inflammatory bowel disease. *World J. Gastroenterol.* **2014**, *20*, 1165–1179, doi:10.3748/wjg.v20.i5.1165.
19. Sochocka, M.; Donskow-Łysoniewska, K.; Diniz, B.S.; Kurpas, D.; Brzozowska, E.; Leszek, J. The Gut Microbiome Alterations and Inflammation-Driven Pathogenesis of Alzheimer's Disease-a Critical Review. *Mol. Neurobiol.* **2019**, *56*, 1841–1851, doi:10.1007/s12035-018-1188-4.
20. Zhang, B.; Wang, H.E.; Bai, Y.M.; Tsai, S.J.; Su, T.P.; Chen, T.J.; Wang, Y.P.; Chen, M.H. Inflammatory bowel disease is associated with higher dementia risk: A nationwide longitudinal study. *Gut* **2020**, *70*, 85–91, doi:10.1136/gutjnl-2020-320789.
21. Hatoum, O.A.; Binion, D.G. The vasculature and inflammatory bowel disease: Contribution to pathogenesis and clinical pathology. *Inflamm. Bowel Dis.* **2005**, *11*, 304–313, doi:10.1097/01.mib.0000160772.78951.61.
22. Schicho, R.; Marsche, G.; Storr, M. Cardiovascular complications in inflammatory bowel disease. *Curr. Drug Targets* **2015**, *16*, 181–188, doi:10.2174/1389450116666150202161500.
23. Singh, S.; Singh, H.; Loftus, E.V., Jr.; Pardi, D.S. Risk of cerebrovascular accidents and ischemic heart disease in patients with inflammatory bowel disease: A systematic review and meta-analysis. *Clin. Gastroenterol. Hepatol.* **2014**, *12*, 382–393.e381, doi:10.1016/j.cgh.2013.08.023.
24. Brudek, T. Inflammatory Bowel Diseases and Parkinson's Disease. *J. Parkinsons Dis.* **2019**, *9*, S331–S344, doi:10.3233/JPD-191729.
25. Franke, A.; McGovern, D.P.B.; Barrett, J.C.; Wang, K.; Radford-Smith, G.L.; Ahmad, T.; Lees, C.W.; Balschun, T.; Lee, J.; Roberts, R.; et al. Genome-wide meta-analysis increases to 71 the number of confirmed Crohn's disease susceptibility loci. *Nat. Genet.* **2010**, *42*, 1118–1125, doi:10.1038/ng.717.
26. Lin, J.C.; Lin, C.S.; Hsu, C.W.; Lin, C.L.; Kao, C.H. Association between Parkinson's disease and inflammatory bowel disease: A nationwide Taiwanese retrospective cohort study. *Inflamm. Bowel Dis.* **2016**, *22*, 1049–1055, doi:10.1097/MIB.0000000000000735.
27. Villumsen, M.; Aznar, S.; Pakkenberg, B.; Jess, T.; Brudek, T. Inflammatory bowel disease increases the risk of Parkinson's disease: A Danish nationwide cohort study 1977–2014. *Gut* **2019**, *68*, 18–24, doi:10.1136/gutjnl-2017-315666.
28. Kosmidou, M.; Katsanos, A.H.; Katsanos, K.H.; Kyritsis, A.P.; Tsivgoulis, G.; Christodoulou, D.; Giannopoulos, S. Multiple sclerosis and inflammatory bowel diseases: A systematic review and meta-analysis. *J. Neurol.* **2017**, *264*, 254–259, doi:10.1007/s00415-016-8340-8.
29. Lin, C.H.; Kadakia, S.; Frieri, M. New insights into an autoimmune mechanism, pharmacological treatment and relationship between multiple sclerosis and inflammatory bowel disease. *Autoimmun. Rev.* **2014**, *13*, 114–116, doi:10.1016/j.autrev.2013.09.011.
30. Harper, J.W.; Zisman, T.L. Interaction of obesity and inflammatory bowel disease. *World J. Gastroenterol.* **2016**, *22*, 7868–7881, doi:10.3748/wjg.v22.i35.7868.
31. Jess, T.; Jensen, B.W.; Andersson, M.; Villumsen, M.; Allin, K.H. Inflammatory Bowel Diseases Increase Risk of Type 2 Diabetes in a Nationwide Cohort Study. *Clin. Gastroenterol. Hepatol.* **2020**, *18*, 881–888.e881, doi:10.1016/j.cgh.2019.07.052.
32. Corlateanu, A.; Mendez, Y.; Wang, Y.; Garnica, R.D.J.A.; Botnaru, V.; Siafakas, N. Chronic obstructive pulmonary disease and phenotypes: A state-of-the-art. *Pulmonology* **2020**, *26*, 95–100, doi:10.1016/j.pulmoe.2019.10.006.
33. Fricker, M.; Goggins, B.J.; Mateer, S.; Jones, B.; Kim, R.Y.; Gellatly, S.L.; Jarnicki, A.G.; Powell, N.; Oliver, B.G.; Radford-Smith, G.; et al. Chronic cigarette smoke exposure induces systemic hypoxia that drives intestinal dysfunction. *JCI Insight* **2018**, *3*, e94040, doi:10.1172/jci.insight.94040.
34. Ribaldone, D.G.; Pellicano, R.; Actis, G.C. The gut and the inflammatory bowel diseases inside-out: Extra-intestinal manifestations. *Minerva Gastroenterol. Dietol.* **2019**, *65*, 309–318, doi:10.23736/S1121-421X.19.02577-7.
35. Young, R.P.; Hopkins, R.J.; Marsland, B. The gut-liver-lung axis: Modulation of the innate immune response and its possible role in chronic obstructive pulmonary disease. *Am. J. Respir. Cell Mol. Biol.* **2016**, *54*, 161–169, doi:10.1165/rcmb.2015-0250PS.
